# Prognostic value of RGS1 and mTOR Immunohistochemical expression in Egyptian multiple myeloma patients; A single center study

**DOI:** 10.1371/journal.pone.0288357

**Published:** 2023-07-12

**Authors:** Nora Hafez, Lobna Refaat, Omnia K. ElGebaly, Hossam M. Elhariry, Mohammed Ghareeb, Lamiaa A. Fathalla

**Affiliations:** 1 Clinical Pathology Department, Ain Shams Specialized Hospital, Ain Shams University, Cairo, Egypt; 2 Clinical Pathology Department, National Cancer Institute, Cairo University, Cairo, Egypt; 3 Medical Oncology Department, National Cancer Institute, Cairo University, Cairo, Egypt; Chung Shan Medical University, TAIWAN

## Abstract

**Introduction:**

Prognostic factors in plasma cell myeloma were proved to be related to signaling pathways and associated transcription factors. RGS1 and mTOR were known to play an important role in the pathogenesis of multiple myeloma. The aim of the study was to evaluate the expression and the prognostic value of RGS1 and mTOR and their relation to clinical as well as other diagnostic criteria in multiple myeloma.

**Patients and methods:**

The present study included 44 denovo Myeloma patients, recruited from the Medical Oncology Department, National Cancer Institute, Cairo University. Detection of RGS1 and mTOR expression was performed using Immunohistochemical staining on bone marrow biopsy sections.

**Results:**

The median age was 51 years with male to female ratio 1.58:1. There was a positive highly statistically significant correlation between RGS1 and mTOR among all studied cases (p value <0.001). Regarding their prognostic value, there was a highly statistically significant association of the expression levels of RGS1 and mTOR with treatment response (p <0.001). Finally, there was a significant influence of RGS1 and mTOR on overall survival probability (p value <0.001 and <0.002 respectively) with better survival for those having low expression.

**Conclusion:**

RGS1 and mTOR were suggested as poor prognostic markers in MM patients, being associated with lower response rate and inferior OS. We recommend considering RGS1 and mTOR as one of the prognostic criteria in different risk stratification and staging classifications. Further trials for RGS1 and mTOR targeting in multiple myeloma are recommended.

## Introduction

Plasma cell myeloma (PCM) is ranked the second most common hematological malignancy worldwide, and in South Africa, PCM is more common in African black population than in the white population with a ratio 2:1 **[1].** In Egypt, multiple myeloma (MM) accounts for 1.3% of all cancers **[2]** and the mean age of the disease is 58.5 years **[3].**

The BM microenvironment (BMM) plays a central role in the pathogenesis of MM **[4],** where it can provide inflammatory agents as cytokines, growth factors like insulin-like growth factor-1 (IGF-1), and others, which support malignant cell growth, drug resistance and cytotoxicity of healthy cells **[5].** Some of the prognostic factors related to the tumor burden are related to the signaling pathways and the associated transcription factors as STAT3 **[6].**

Regulator of G proteins signaling (RGS) proteins are a member family of intracellular regulatory proteins **[7],** that are expressed on hematopoietic cells such as T and B lymphocytes **[8],** natural killer cells **[9],** and dendritic cells **[10].** RGS1 is known to negatively regulate heterotrimeric G protein-coupled receptor (GPCR) signaling pathways, by accelerating intrinsic GTPase activity **[11]** of G protein α subunits, favoring their inactivation by reassociation with the βγ dimer and thereby inhibiting their downstream activity **[12].** On the other hand, an important mechanism of action of RGS1 is the non-GAP signaling, where RGS1 promotes malignancy progression by the competitive action with Gβγ for Gα binding, due to the similarity of the two surfaces of Gα that interact with both Gβγ and RGS proteins. This favors the occurrence of some downstream effects of both Gβγ to Gαs subunits **[13].**

Generated Gα and Gβγ initiate downstream signaling networks by interacting with several effector molecules as phospholipase C (PLC), phosphoinositide 3- kinase (PI3K), **[14],** and increasing the activity of mitogen-activated protein kinase (MAPK) signaling pathways through PI3K/Akt and PLCβ. Also, this occurs through interaction with insulin-like growth factor receptor 1 (IGFR1) and c-Src tyrosine kinase **[15]**. All these factors will promote survival, differentiation, and proliferation of the cells and provide higher resistance to apoptosis **[16].**

Many studies highlighted the importance of RGS in the autoimmune diseases as multiple sclerosis, Crohn’s disease, ulcerative colitis **[17],** and undifferentiated spondylo-arthritis, often associated with gastrointestinal lesions **[18].** Also, RGS1 gene amplification was detected in various malignancies, including melanoma, non-Hodgkin lymphoma, retinoblastoma, pancreatic cancer, nasopharyngeal carcinoma **[19],** diffuse large B-cell lymphomas, follicular lymphomas, and multiple myeloma **[11]. Roh et al.** suggested that RGS1 protein may be a promising prognostic marker for MM risk stratification and a promising target for a new MM therapy as well **[11].**

The mTOR (mechanistic target of rapamycin) is a serine/threonine protein kinase that is expressed throughout the whole body **[20]**. mTOR signaling is generally involved in regulating cell survival, growth, metabolism, protein synthesis and autophagy, as well as homeostasis **[21].** This signaling pathway is critical in normal hematopoietic cells’ development and function **[22]**, which occurs through regulation of some proteins such as STAT3 **[23].** Additionally, mTORC1 (mTOR complex 1) is regulated by several signaling pathways including the PI3K/Akt pathway, the Ras-MAPK pathway, and some other intracellular factors **[23,24].** Insulin is the most-characterized growth factor (IGF) that activates mTORCs through the PI3K/Akt/TSC/Rheb pathway **[25].**

Deregulated mTOR was found to be associated with human growth and metabolic diseases as neuronal degeneration, obesity, and type 2 diabetes **[26].** Several studies reported that mTOR is aberrantly overactivated in more than 70% of cancers and mTOR dysfunction contributes to tumorigenesis **[23]** as in kidney cancer **[27],** breast cancer, non-small cell lung cancer (NSCLC) **[28]** and multiple myeloma **[29,30].**

mTORC is rapamycin sensitive **[31].** Accordingly, Rapalogs (rapamycin and its analogs) was found to induce inhibit the tumor progression by inducing tumor cell apoptosis, cell cycle arrest and signal transduction inhibition in many hematological diseases, one of them is multiple myeloma **[22,32,33].**

The aim of the study was to evaluate the expression of RGS1 and mTOR markers and their relation to the clinical and diagnostic parameters as well as their prognostic value in MM. To the best of our knowledge, this study is one of the few studies that have been done to evaluate the IHC expression of RGS1 and mTOR in BM biopsies of MM patients **[11,30,34]** and the first study to highlight the role of these markers in Egyptian multiple myeloma patients.

## Methods

The present study included 44 de-novo Egyptian Multiple Myeloma cases, above 18 years, attended to the outpatient clinic in the National Cancer Institute (NCI), Cairo University, throughout 18 months. All patients were diagnosed ant treated in the NCI **(S1 Appendix and S2 Appendix respectively).**

This study was approved by the ethical committee, review board of National Cancer Institute, Cairo University in accordance with Helsinki guidelines for the protection of human subjects. Written informed consent was obtained from all patients (IRB no. IRB00004025).

**Fig 1:**
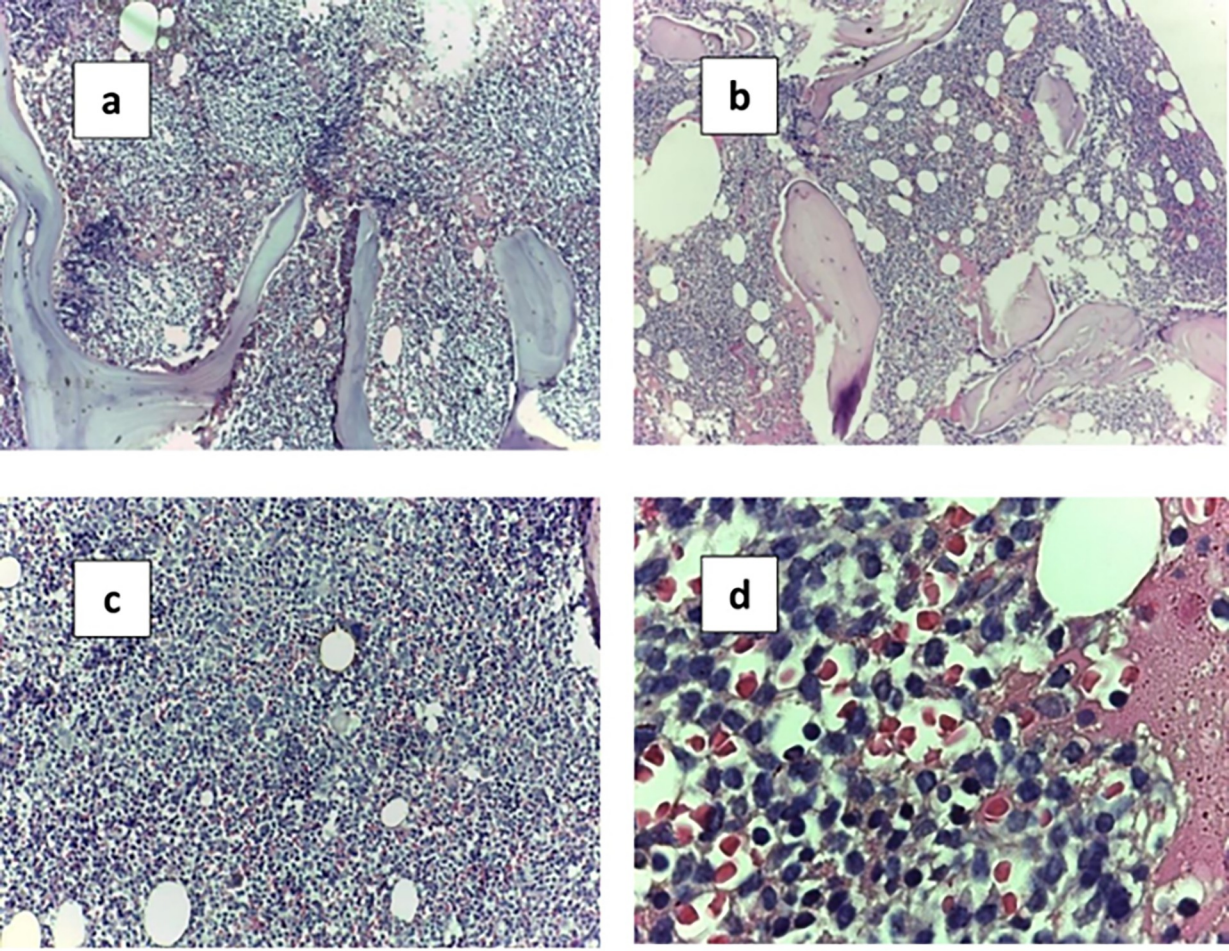
BMB H&E sections with plasma cells infiltration. A. Para trabecular and patchy infiltration by plasma cells with power magnification (10x). B. Interstitial infiltration by plasma cells with power magnification (10x). C. Interstitial infiltration by plasma cells with power magnification (40x). D. Plasma cells infiltration power magnification (100X Oil emersion lens).

The BM trephine biopsy was performed at diagnosis by using trephine biopsy needles from the posterior superior iliac crest. Biopsy cores were prepared and stained for microscopic examination **(Fig 1).**

**Fig 2:**
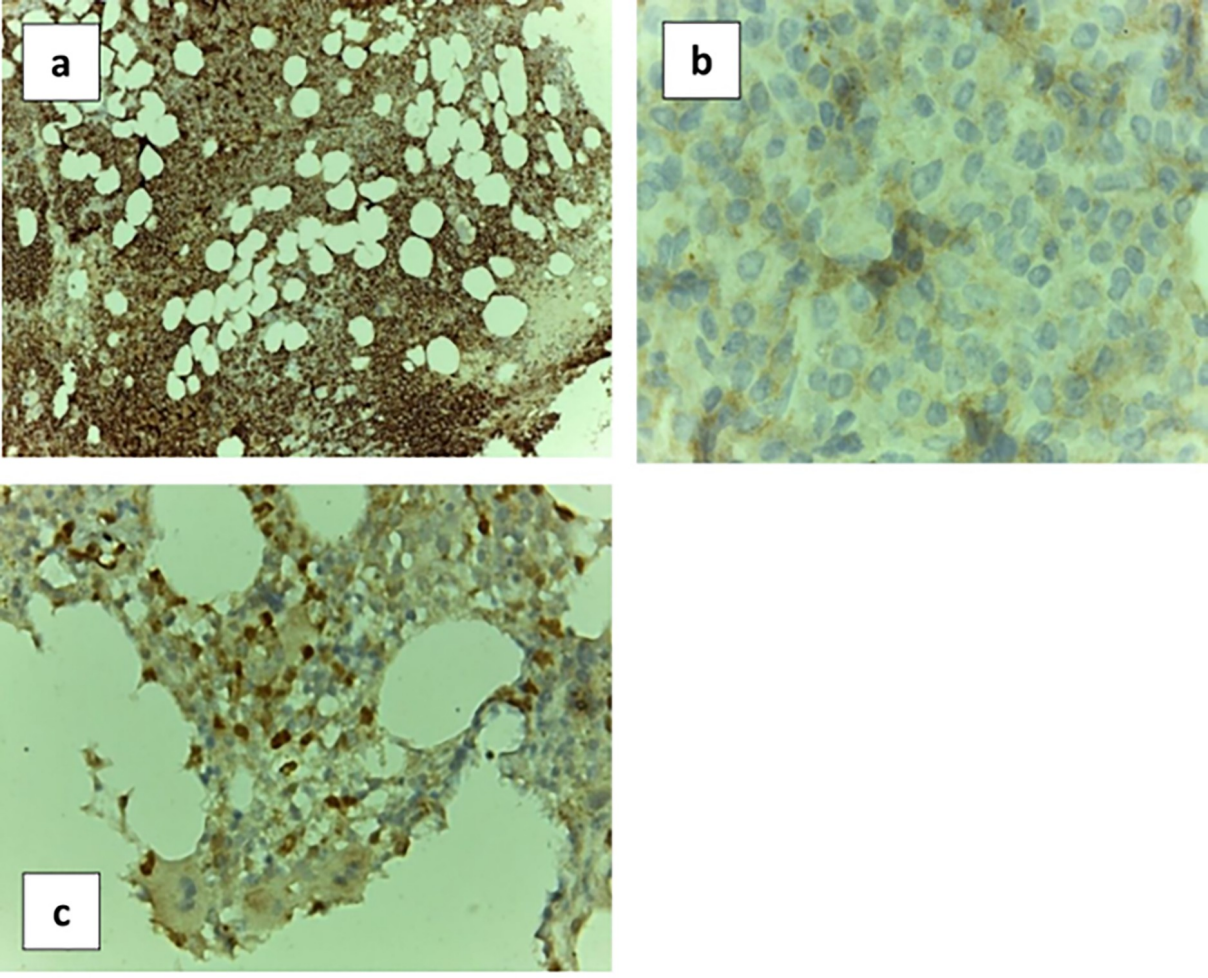
BMB IHC sections with plasma cells infiltration. A. Positive immunohistochemical staining CD138 with power magnification (10x). B. Positive immunohistochemical staining mTOR (100x). C. Positive immunohistochemical staining RGS1 (40x).

Immunohistochemical (IHC) staining was done by monoclonal Mouse IgG Anti-Human **CD138** Clone (MI15 Ready-to-Use (Dako Omnis) Code GA642), monoclonal rabbit IgG Anti-Human **mTOR** (7C10, #2983, from Cell Signaling Technology, Beverly, MA) (1:250), and polyclonal rabbit IgG Anti-Human **RGS1** protein (NBP1-68645, Novus Biologicals, Littleton, Colorado, USA) (1:250) **(Fig 2).**

**Interpretation of the immunohistochemical stain analysis.** Analysis of whole tissue section slides under an Olympus light microscope was performed and histopathological features were evaluated. Plasma cell ratio in bone marrow was confirmed by correlation with the PC counts in Giemsa-stained bone marrow aspirations and CD138 IHC to confirm the presence and actual percentage of plasma cells.

Immunostaining for mTOR was cytoplasmic. IHC was scored with regards to both the extent and intensity of staining. The intensity was graded from 0 to 2 (‘0’ for absent staining, ‘1’ for dim expression, and ‘2’ for moderate to strong expression). The percentage of tumor with positive staining was graded from 1 to 3 (“1” represents <10% tumor positivity, “2” represents 10–50% tumor positivity, and “3” for > 50% positivity). For comparison with clinical variables, the expression level of the biomarker was semi-quantitated by multiplying the scores of the staining intensity and the percentage of positive tumor **[30].** Then we classified the patients into two groups according to the best scoring level by using Receiver operating characteristics (ROC) curve analysis. Correlations of mTOR to clinical variables and other investigations were done.

Regarding RGS1, three major RGS1 IHC expression patterns were observed: negative, predominantly cytoplasmic and both cytoplasmic and nuclear. Subsequently, the relative percentage of simultaneous cytoplasmic and nuclear RGS1-positive cells were counted and analyzed relative to the overall number of tumor cells **[11]**. The optimal cut-off value of RGS1 IHC was evaluated using a ROC curve analysis and correlations were done with different investigational and clinical variables.

Data was analyzed using IBM SPSS statistics (V. 26.0, IBM Corp., USA, 2019) **(S3 Appendix).**

## Results

The demographic, clinical and laboratory data of the studied groups are represented in **Table 1.** The studied cases were 27/44 males (61.4%) and 17/44 females (38.6%) with male to female ratio 1.58:1. Their age ranged from 25 to 82 years with a mean value 52.23 ± 11.06 and a median value 51. The cut off levels of defining end organ damage were set according to the updated IMWG criteria 2016 **[35]**.

**Table 1:** Demographic, clinical and laboratory data of the studied groups.

	Number = 44 (%)
**Age**	
median(range)	51 (25–82)
**Gender**	
Female	27 (61.4%)
Male	17 (38.6%)
**CRAB criteria**	
**Hemoglobin (n = 44)**	
< 10 g/dl	35 (79.5)
≥ 10 g/dl	9 (20.5)
median(range)	8.65 (5.3–16)
**Serum Ca (n = 44)**	
≤ 11 mg/dl	33(75.0)
> 11 mg/dl	11(25.0)
median(range)	9.4 (3.9–14.3)
**Serum Creatinine (n = 44)**	
≤ 2 mg/dl	27 (61.4)
> 2 mg/dl	17 (38.6)
median(range)	1.2 (0.4–7.8)
**Osteolytic bony lesions (n = 44)**	
Negative	6 (13.6)
Positive	38 (86.3)
**Other Biochemical laboratory profile**	
**β2 microglobulin**	
< 3.5 mg/l	1 (2.3)
**≥ 3.5** mg/l	97.7)
**Albumin (gm/dl)**	
median(range)	3 (2–9)
**LDH (IU/L)**	
median(range)	310 (114–1045)
**Bone marrow examination**	
**BMA plasma cells (%)**	
median(range)	30 (15.0–84.0)
**BMB CD 138 (%)**	
median(range)	80 (20–100)
**BMB pattern of distribution**	
Diffuse	28 (63.6)
Interstitial	16 (36.4
**Serum protein electrophoresis and immunofixation**	
IgA Kappa	5 (11.4)
IgA lambda	5 (11.4)
IgG kappa	22 (50.0)
IgG lambda	12 (27.3)
**Free light chain (FLC)**	
K-FLC	3 (6.8)
L-FLC	5 (11.4)
No FLC	36 (81.8)
**Response to treatment (n = 44)**	
Responders	16 (36.4)
Non-responders	28 (63.6)

Plasma cells were examined from BMA smears and the percentage ranged from 15% to 84%. Immunohistochemistry CD138 showed increased levels in all cases, correlating with the PC counts. The % of CD138 positivity ranged from 20% to 100% infiltration with a diffuse or interstitial pattern of infiltration. M band was detected in SPE in all patients. Immunofixation revealed either IgA or IgG bands. Free light chain was present in 8/44 patients (18.2%).

### Immunohistochemical staining of RGS1 and mTOR in MM patients

#### A. RGS1 and mTOR expression distribution in MM patients (n = 44)

RGS1 expression was estimated in all the patients and the % of expression ranged from 0.0% to 100% with a mean value 46.57 ± 23.88 and a median value 50%. As regards the mTOR expression, every patient had a different score level according to intensity and % of expression. Accordingly, the patient fell into 1 of 7 categories from score 0 to 6 as shown in **Table 2.**

**Table 2:** RGS1 and mTOR expression distribution in the studied patients.

Marker expression	Number = 44 (%)
**RGS1**	
>35%	28 (63.6)
≤35%	16 (36.3)
Median (range)	50 (0.0–100.0)
**mTOR score level**	
0	7 (15.9)
1	10 (22.7)
2	5 (11.3)
3	11(25.0)
4	3 (6.8)
5	0 (0.0)
6	8 (18.2)

#### B. Receiver operating characteristics (ROC) curve for determination of the best cut-off values for RGS1 and mTOR

**Fig 3:**
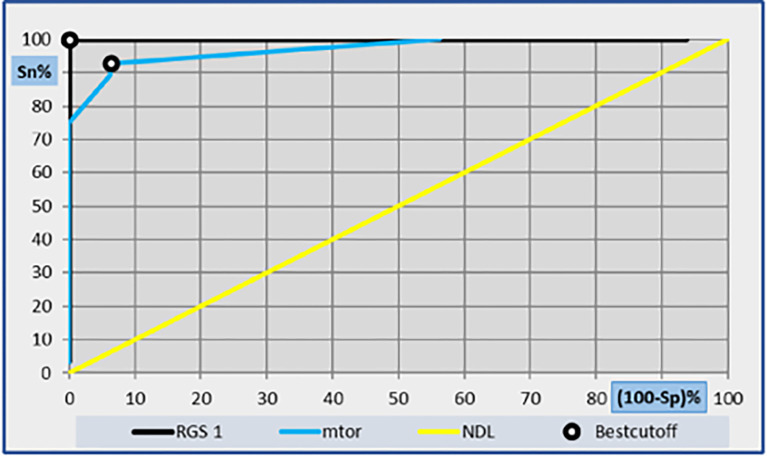
ROC curve analysis showing the diagnostic performance of RGS1 and mTOR for discriminating of the best cutoff value.

ROC curve was done for calculation of RGS1 and mTOR cut offs as regards sensitivity and specificity with 95% confidence interval. As regards RGS1, the area under the ROC curve (AUC) was 0.938 with the best cut-off value 35% with 100% specificity and 100% sensitivity. As regards mTOR, the AUC was 0.533 and the best cut-off score value was 1 with 93.8 specificity and 92.9 sensitivity **as shown in Fig 3.**

### C. Comparative analysis of RGS1 and mTOR with the studied patients

#### Association between RGS1 expression at cutoff value 35% and MM patients as regarding demographic & laboratory data

There was a **highly statistically significant** association between RGS1 expression and LDH levels. Where, 13/16 (81.3%) of those having low RGS1 were having low LDH levels as well. While 26/28 (92.9%) of those having high RGS1 expression were having high LDH levels as well (P <0.001). OR = 56.3 (95CI: 8.35–380.1); Significant positive risk (Table 3).

**Table 3:** The association of RGS1expression and other parameters.

Clinicopathological features		RGS1		Total No. (%)	P value
		≤ 35 (N = 16)	>35 (N = 28)		
		No. (%)	No. (%)		
**LDH *(IU/L)***	**≤ 250**	13 (81.3)	2 (7.1)	15 (34.1)	**<0.001** *
	**>250**	3 (18.8)	26 (92.9)	29 (65.9)	
**Clinicopathological features**		**mTOR score level**		**Total** **No. (%)**	**P** **value**
		**> 1 (n = 27)**	**≤ 1 (n = 17)**		
		**No. (%)**	**No. (%)**		
**LDH *(IU/L)***	**≤ 250**	2 (7.4)	13 (76.5)	15 (34.1)	**<0.001** **
	**>250**	25 (92.6)	4 (23.5)	29 (65.9)	
**FLC**	**Kappa-LC**	2 (5.4)	1 (14.3)	3 (6.8)	**0.074** **
	**Lambda-LC**	4 (10.8)	1 (14.3)	5 (11.4)	
	**No FLC**	31 (83.8)	5 (71.4)	36 (81.8)	

** P-value < 0.05: Significant; P-value < 0.01: Highly significant (Chi-square test).

* OR = 56.3 (95CI: 8.35–380.1); Sig. Pos. Risk.

On the contrary, there was no statistically significant association between RGS1 expression and demographic and other chemical laboratory data **(S4 Table (1) in S4 Appendix).**

#### Association of mTOR expression at cutoff score value 1 and MM patients regarding demographic & laboratory data

There was a **highly statistically significant** association between mTOR expression and LDH levels. Where, 25/27 (92.6%) patients of those having mTOR expression level >1 were having high LDH levels as well. While 13/17 (76.5%) patients of those having expression level of mTOR ≤1 were having low LDH levels as well **(P <0.001).** On the contrary, there was no statistically significant association between mTOR expression and other demographic and laboratory data) **(S4 Table (2) in S4 Appendix).**

Moreover, there was **a borderline statistically significant** association between mTOR expression and FLC (P value = 0.074). While there was no statistically significant association between mTOR expression and SPE (P value = 0.597).

#### Association of RGS1 and mTOR expression and response to treatment

There was a **highly statistically significant** association between the expression of RGS1 as well as mTOR expression and the response to treatment among our patients (Table 4).

**Table 4:** Association of RGS1 expression and mTOR score level and response to treatment.

Response	RGS1		P value
	≤ 35 no = 16 (%)	>35 no = 28 (%)	
**Responders**	**16 (100)**	**0 (0.0)**	**<0.001** *
**Non responders**	**0 (0.0)**	**28 (100)**	
	**mTOR score level**		
	**>1** **no = 27 (%)**	**≤1** **no = 17 (%)**	
**Responders**	**1 (3.7)**	**15 (88.2)**	**<0.001** *
**Non responders**	**26 (96.3)**	**2 (11.8)**	

*p-value < 0.01: Highly significant (Chi-square test).

#### The relation between mTOR and RGS1 expression in MM patients

There was a **positive highly statistically significant** correlation between mTOR expression and RGS1 expression among all studied cases (P-value <0.001) (Table 5).

**Table 5:** The relation between RGS1 and mTOR expression in MM patients.

			RGS.1		Total	P value
			≤35	>35		
**mTOR score level**	**>1**	**Count (%)**	1 (3.7)	26 (96.3)	27 (61.3)	**<0.001** *
	**≤1**	**Count (%)**	15 (88.2)	2 (11.8)	17 (38.6)	
**Total**		**Count (%)**	16 (100)	28 (100)	44 (100)	

*P-value < 0.01: Highly significant (Chi-square test).

### D. Survival analysis; overall survival (OS)

Median follow up time of the studied group was 20.5 months ranging from 6 month to 44 months. Overall survival probability was estimated for the whole group; and the survival estimate at 1 year was 38.6% and at 2 years was 38.6% with a median estimate of 20.5 months.

#### Relationship between OS and RGS1 as well as mTOR expression (Fig 4)

Results revealed that there was a significant influence of RGS1 expression and mTOR expression on overall survival probability **(p value 0.001 and 0.002 respectively)** with better survival for those having low expression.

**Fig 4:**
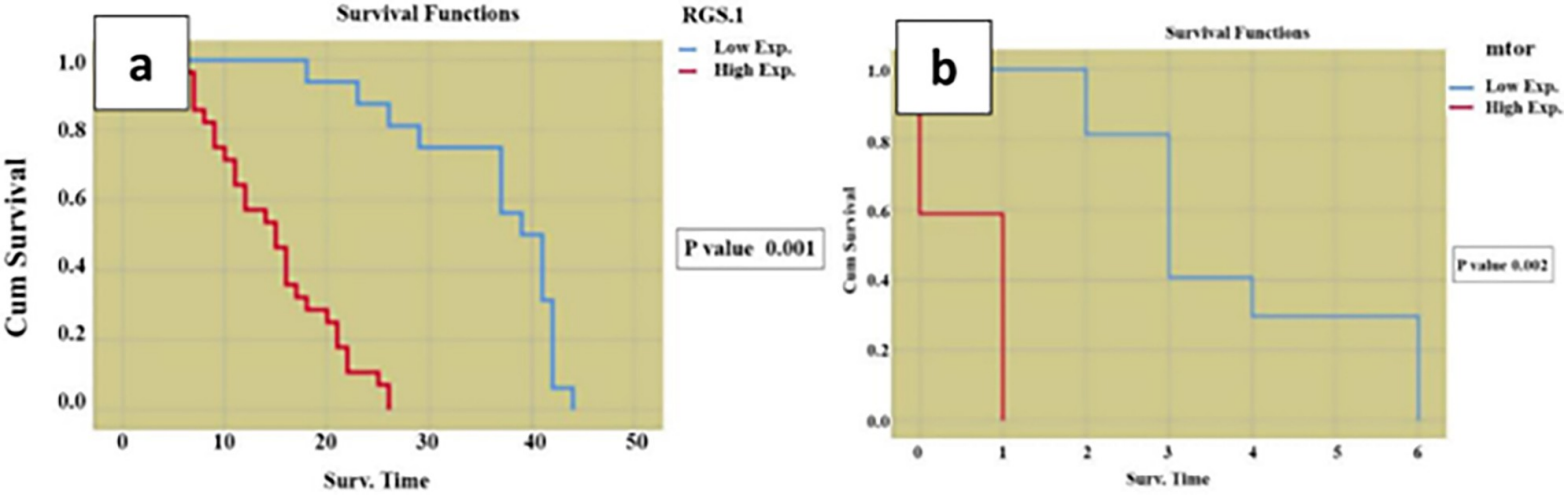
Kaplan- Meier analysis for the relation between (a) RGS1 and (b) mTOR results and overall survival among the studied.

### Relationship between OS and different studied parameters

P value of the relationship between demographic, clinical and laboratory data with the OS **(S4 Table (3) in S4 Appendix).** Results showed that there was a statistically significant difference in the OS among MM patients in our study and LDH with a better overall survival for those having LDH level < 250 IU/L **(p = 0.003).** Multivariate analysis was done for the statistically significant variables in the univariate level **as shown in S4 Table (4) in S4 Appendix**, it revealed that RGS1 and mTOR remained an independent prognostic factors of the OS in MM patients with **p value = 0.05 and p value = 0.01 respectively.** However, LDH lost its prognostic significance in the OS.

## Discussion

MM is characterized by a wide heterogeneity in outcome ranging from few months to more than 10 years **[36]**. For this reason, various prognostic factors and staging systems have been developed trying to predict the outcome of the disease **[37].** The present study included 44 de-novo Multiple Myeloma cases with male to female ratio 1.58:1. Their age ranged from 25 years till 82 years with a median age 51 years. This prevalence is nearly like previous studies done in Egyptian multiple myeloma patients, where the mean age was 58.5 with a male to female ratio 1.5:1 and like some previous studies as well **[3,38–40]**. Most of our patients usually visit the clinics in advanced stage. So, our results reflect a more advanced stage according to IMWG, ISS and mSMART staging systems **[41,42]**.

Several recent reviews have described the multiple roles of GPCR signaling in cancer **[43].** RGS1 was shown to have a role in the proliferation, invasion, and metastasis of malignancies **[44].** Previous studies have reported the upregulation of RGS1 expression in MM **[11,45]**. Also, mTOR shares some signaling pathways with RGS1 as the PI3K/Akt cell cycling pathway, which contributes to the initiation and maintenance of cancer as in Non-Hodgkin lymphomas **[46,47]**. Review of literature reveals a few studies on MM cell lines to study the dysregulation of m-TOR pathway **[22,48],** and nowadays, mTOR signaling has become a target for treatment to increase overall survival **[29]**.

RGS1 expression was estimated in all the patients and the % of expression ranged from 0.0% to 100% with a median value 50%. The cutoff value was chosen by using ROC curve. High RGS1 expression (>35%) was detected in 28/44 patients (63.6%), while 16/44 patients (36.3%) showed low expression (≤35%). In a study done by ***Roh et al*.,** to study the RGS1 expression in multiple myeloma BMB sections, they classified their patients into high versus low expression with a cutoff value 7 which is equivalent to 60%-70% expression, they found that this is the significant cutoff value in their population **[11].** This may be related to the larger number of patients in their study and ethnic variation between the Asian and Egyptian populations. Also, 59.5% of their studied group was classified as a standard risk category according to IMWG risk stratification, while our study included all stages of MM.

RGS1 expression showed a highly statistically significant association with LDH levels. Also, ***Roh et al*.,** found a borderline correlation between RGS1 expression and LDH levels **(p value = 0.060) [11].** This may be related to the advanced stage of the disease in our studied population. On the contrary, there is no statistically significant association between RGS1 expression and other demographic and laboratory data. Moreover, there was no statistically significant association between RGS1 expression and neither BMA plasma cells percentage, nor BMB CD 138 nor BMB infiltration pattern. Similar results were found in ***Roh et al*. [11].**

As regards mTOR expression, in accordance with ***Stockwin et al*.,** patients were categorized into 7 scores according to the mTOR expression as regards the intensity of the marker and the percentage of expression. Patients with score level 6 in our study were 18.7% compared to the 25.8% in ***Stockwin et al*.,** however, ***Stockwin et al*.** performed IHC staining with mTOR and phospho-mTOR (p-mTOR) on multiple myeloma BMB sections, but in our study, we studied only the mTOR **[30].** So, this may reflect a relative higher expression of the marker in their study. Accordingly, in our study, a ROC curve was done and score level 1 was chosen. According to the scoring system, 17/44 (38.6%) patients were having a score level ≤1 and 27/44 (61.4%) were having a score level >1.

Near to our results, ***Sebestyen et al*.** verified that mTOR activity was presented among DLBCL patients in 62% of the studied population, by positive immunostaining for p-S6 the most sensitive marker of mTOR activity **[49].** Also, ***Schedel et al*.,** examined the expression of mTOR and its phosphorylated (active) counterpart found **[50].** The results suggested that mTOR is highly active in different cancer types, as various permanent cell lines of bladder cancer and head & neck squamous cell carcinoma.

Like RGS1, mTOR showed a highly statistically significant association with LDH levels. This may be related to the advanced stage of the disease. Also, there was a borderline statistically significant association with FLC assay. On the contrary, there is no statistically significant association with other parameters.

Like our results, ***Stockwin et al*.** found no significant association with the age of their patients **[30].** However, they found a significant correlation between mTOR expression and male gender in the MM patients (p = 0.04) and ***Vajpayee et al*.** *found* a significant correlation between mTOR expression and male gender in diffuse large B-cell lymphoma (DLBDL) patients (85% vs. 46%, p < 0.01) **[46].** However, the former study showed a male to female ratio 1:0.72 and the latter a male to female ratio 1.89:1 and this is different from our studied population ratio which is 1.58:1.

Regarding the response to treatment, there was a **highly statistically significant** association between the expression of both RGS1 and mTOR with the response to treatment among our patients. This can be explained by the poor prognostic association between RGS1 and different cancer diseases, supporting the proliferation and invasion of cancer cells ^**(11,46,53)**.^ ***Vajpayee et al*.** studied the mTOR marker among the diffuse large B cell lymphoma patients and revealed that the high mTOR expression showed a trend toward advanced clinical stage than others (78% vs. 54%, p = 0.08) **[46].**

Also, there was highly **statistically significant positive association** between RGS1 and mTOR expression in MM patients, and both markers showed inferior prognostic significance in MM. Where, both RGS1 and mTOR showed an inferior overall survival influence in MM, with better survival for those having low marker expression levels. Both RGS1 and mTOR remained an independent prognostic factor of the OS in MM patients after multivariate analysis.

Like our results, RGS1 protein expression was significantly associated with unfavorable prognosis of the patients in DLBCL **[51]** and in melanoma **[44]**. Also, similar mTOR results were found in previous studies among DLBCL patients **[46,49]**.

This association and prognostic significance can be explained by sharing some receptors and common signaling pathways by both RGS1 and mTOR, that are involved in the pathogenesis of MM as well, like IGFR1, PIK3/AKT, MAPK **[52–54]**. So, higher expressions may be associated with hyperactivity in the signaling pathways and myeloma progression as well.

Gβγ, which is activated by RGS1, has been shown to increase the activity of MAPK signaling pathways through PI3K/Akt and PLCβ, as well as through IGFR1 **[53]**. Similarly, the mTORC is regulated by the IGF, PI3K/Akt/mTOR pathway, the Ras-MAPK pathway, and some other intracellular factors **[25,55–57]**.

Akt, which is commonly found to be hyperactive in cancers, is an important substrate of mTORC **[58]**. The cytokines secreted in BMM which are contributed to the pathogenesis of MM as IL6, VEGF and IGF activate their respective receptors expressed on MM cells through an important pathway, PI3K/AKT/mTOR, and promote tumor growth as well as resistance development to existing therapies **[59,60].**

Since RGS1 & mTOR were shown to play a significant role in disease pathogenesis, they may be a new target for immunotherapy in many diseases. RGS1 was shown to be a novel target for inflammatory diseases as Rheumatoid arthritis [**61]**, melanoma **[62]** and cervical cancer **[63]**.

Regarding mTOR, Rapamycin was the first mTOR inhibitor, and was initially used as an immunosuppressive drug in the field of solid organ transplantation **[33]**. Moreover, there was a strong rationale for mTOR inhibitors (mTORi) effects in many hematological diseases due to the associated hyperactivation of mTOR, as in acute leukemia, lymphoma, multiple myeloma, Waldenström macroglobulinemia, and GVHD **[22,32]**. However, preclinical trials are needed to confirm these findings.

## Conclusion

RGS1 and mTOR were found to be poor prognostic markers in MM patients, being associated with lower response rate and inferior OS. Further studies should be done on larger population to assess the association with other prognostic markers. From these findings, we could recommend considering RGS1 and mTOR as one of the prognostic criteria in the different risk stratification and staging classifications. Further trials for RGS1 and mTOR targeting in multiple myeloma are recommended.

## Limitations

The study enrolled a relatively small number of cases which reduced the capability to identify certain associations.

## Supporting information

S1 Appendix Diagnosis of the patients. (DOCX)

S2 Appendix Treatment regimen. (DOCX)

S3 Appendix Statistical methods. (DOCX)

S4 Appendix Tables: • S4 Table (1): The association of RGS1expression and other parameters
• S4 Table (2): The association of mTOR and other parameters
• S4 Table (3): Overall Survival (OS) of the studied patients and its relation to other prognostic factors
• S4 Table (4): Multivariate analysis for OS (cox regression model)
(DOCX)

## Abbreviations

ABMT: autologous bone marrow transplant
AC: adenylyl cyclase
AUC: area under the curve
BMM: Bone marrow microenvironment
CR: complete response
GPCR: G protein-coupled receptor
IGF: insulin growth factor
IGF-1: insulin-like growth factor-1
IHC: Immunohistochemistry
LDH: lactate dehydrogenase
MAPK: mitogen-activated protein kinase
MM: multiple myeloma
mTOR: mechanistic target of rapamycin
NSCLC: non-small cell lung cancer
PCM: Plasma cell myeloma
PI3K: phosphoinositide 3- kinase
PLC: phospholipase C
Rapalogs: rapamycin and its analogs
RGS: Regulator of G proteins signaling
ROC: receiver operating characteristics
