# Supplementary material for: Prognostic value of RGS1 and mTOR Immunohistochemical expression in Egyptian multiple myeloma patients; A single center study
Source: PLoS One. 2023 Jul 12;18(7):e0288357. doi: 10.1371/journal.pone.0288357 (PMC10337974; doi:10.1371/journal.pone.0288357)
Supplement: S1 Appendix — (DOCX) [file pone.0288357.s001.docx]

**S1 Appendix: Diagnosis of the patients**

**All patients were confirmed to have multiple myeloma by** personal history, clinical examination and radiological investigations including bone X-ray, CT and/or MRI. Routine diagnostic workup for MM included complete blood count & microscopic examination of Leishman-stained peripheral blood smear, biochemical profile including serum calcium, creatinine, serum albumin, lactate dehydrogenase (LDH) and β_2_-microglobulin and detection and evaluation of monoclonal antibody by serum protein electrophoresis, serum immunofixation.

BM aspirate smears and biopsy were done for all the patients. Minimum accepted core length was at least 2 cms. Touch imprint was made from the BM core prior to placing in fixative. Histological sections were prepared from fixed biopsy specimens which have been decalcified and paraffin - embedded. Sections (3-4μm thick) were cut and used for hematoxylin and eosin, and Immunohistochemistry (IHC)
