# Supplementary material for: Prognostic value of RGS1 and mTOR Immunohistochemical expression in Egyptian multiple myeloma patients; A single center study
Source: PLoS One. 2023 Jul 12;18(7):e0288357. doi: 10.1371/journal.pone.0288357 (PMC10337974; doi:10.1371/journal.pone.0288357)
Supplement: S2 Appendix — (DOCX) [file pone.0288357.s002.docx]

**S2 Appendix: Treatment regimen**

All the patients were offered Bortezomib (Velcade) based regimen (VCD protocol) with the following weekly schedule: Cyclophosphamide 300/m^2^ mg IV, Bortezomib 1.5 mg/m^2^ is taken as intravenous bolus infusion over 3-5 seconds, and Dexamethasone 40 mg is administered orally or IV.

Reassessment clinically, SPEP with immunofixation were requested monthly and BMA every 12-16 weeks. Patients who achieved complete response (CR) criteria were referred for autologous bone marrow transplant (ABMT) (if they are transplant-eligible then they may be offered maintenance lenalidomide according to risk). **Complete response** **was defined as** negative immunofixation on serum and urine samples, disappearance of any soft tissue plasmacytomas, and <5% plasma cells in the bone marrow **^[1]^.**

Patients with relapsed/refractory disease were offered 2^nd^ line according to their performance status, comorbidities, renal & hepatic functions. Examples of 2^nd^ line therapy included lenalidomide +/- dexamethasone, VAD regimen, melphalan and DECP regimen. Some patients were offered ABMT after successful salvage 2^nd^ line. **Relapse was considered if any one or more of the following criteria was fulfilled:** reappearance of serum or urine M-protein by immunofixation or electrophoresis, development of ≥5% plasma cells in the bone marrow, appearance of any other sign of progression (ie, new plasmacytoma, lytic bone lesion, or hypercalcemia) **^[2]^**.

**References:**

1. Kyle RA and Rajkumar SV (2009) Criteria for diagnosis, staging, risk stratification and response assessment of multiple myeloma. Leukemia.; doi.org/10.1038/leu.2008.291.
2. Sonneveld P, Broijl A (2016) Treatment of relapsed and refractory multiple myeloma. Haematologica.; doi: 10.3324/haematol.2015.129189.
