# Supplementary material for: Prognostic value of RGS1 and mTOR Immunohistochemical expression in Egyptian multiple myeloma patients; A single center study
Source: PLoS One. 2023 Jul 12;18(7):e0288357. doi: 10.1371/journal.pone.0288357 (PMC10337974; doi:10.1371/journal.pone.0288357)
Supplement: S3 Appendix — (DOCX) [file pone.0288357.s003.docx]

**S3 Appendix: Statistical methods**

Data was analyzed using IBM SPSS statistics (V. 26.0, IBM Corp., USA, 2019). Numerical data was described as median and range or mean and standard deviation as appropriate, while qualitative data were described as number and percentage. Chi-square test was done to study the association between each 2 variables or comparison between 2 independent groups as regards the categorized data. The probability of error at 0.05 was considered sig., while at 0.01 and 0.001 were highly significant. Diagnostic validity tests were done for calculating the diagnostic sensitivity, the diagnostic specificity, the predictive value for a positive test, the predictive value for a negative test, and the efficacy or the diagnostic accuracy of the test.

The ROC was constructed to obtain the most sensitive and specific cutoff for each technique and AUC was calculated. Calculated Odd’s Ratio, that measures how many times the risk was present among diseased individuals as that among non-diseased ones, were calculated as absolute figures and as a standard error of estimate (95P). Survival analysis was done using Kaplan-Meier method. Median survival time and log-rank were used for comparison between two survival curves. Overall survival (OS) was calculated from date of diagnosis till date of death or last follow up. P-value ≤0.05 was considered significant and all tests were 2 tailed.
