# Supplementary material for: Prognostic value of RGS1 and mTOR Immunohistochemical expression in Egyptian multiple myeloma patients; A single center study
Source: PLoS One. 2023 Jul 12;18(7):e0288357. doi: 10.1371/journal.pone.0288357 (PMC10337974; doi:10.1371/journal.pone.0288357)
Supplement: S4 Appendix — Tables: • S4 Table (1): The association of RGS1expression and other parameters • S4 Table (2): The association of mTOR and other parameters • S4 Table (3): Overall Survival (OS) of the studied patients and its relation to other prognostic factors • S4 Table (4): Multivariate analysis for OS (cox regression model) (DOCX) [file pone.0288357.s004.docx]

**S4 Table (1): The association of RGS1expression and other parameters:**

| **Clinicopathological features** | | **RGS1** | | **Total**  **No. (%)** | **P**  **value** |
| --- | --- | --- | --- | --- | --- |
|  |  | **≤** **35 (N= 16)** | **>35 (N= 28)** |  |  |
|  |  | **No. (%)** | **No. (%)** |  |  |
| ***Demographic data*** | | | | | |
| **Sex** | **Females** | 7 (43.7) | 10 (35.7) | 17 (38.6) | 0.598 |
|  | **Males** | 9 (56.3) | 18 (64.3) | 27 (61.4) |  |
| **Age *(years)*** | **≤ 35** | 9 (56.3) | 16 (57.1) | 25 (56.8) | 0.954 |
|  | **>35** | 7 (43.8) | 12 (42.9) | 19 (43.2) |  |
| ***Hematological data*** | | | | | |
| **HB *(gm/dl)*** | **< 10** | 13 (81.3) | 22 (78.6) | 35 (79.5) | 0.832 |
|  | **≥ 10** | 3 (18.8) | 6 (21.4) | 9 (20.5) |  |
| **BMA plasma cells (%)** | **≤ 36** | 9 (56.3) | 20 (71.4) | 29 (65.9) | 0.307 |
|  | **>36** | 7 (43.8) | 8 (28.6) | 15 (34.1) |  |
| **BMB CD 138 *(%*)** | **≤80** | 10 (62.5) | 14 (50.0) | 24 (54.5) | 0.423 |
|  | **>80** | 6 (37.5) | 14 (50.0) | 20 (45.5) |  |
| **BMB pattern of distribution** | **Diffuse** | 11 (68.8) | 17 (60.7) | 28 (63.6) | 0.594 |
|  | **Interstitial** | 5 (31.3) | 11 (39.3) | 16 (36.4) |  |
| ***Biochemical profile*** | | | | | |
| **Creatinine *(mg/dl)*** | **≤ 2** | 10 (62.5) | 17 (60.7) | 27 (61.4) | 0.907 |
|  | **>2** | 6 (37.5) | 11 (39.3) | 17 (38.6) |  |
| **Ca *(mg/dl)*** | **≤ 11** | 13 (81.3) | 20 (71.4) | 33 (75.0) | 0.469 |
|  | **>11** | 3 (18.8) | 8 (28.6) | 11 (25.0) |  |
| **Albumin *(gm/dl)*** | **≤ 3.5** | 12 (75.0) | 16 (57.1) | 28 (63.6) | 0.236 |
|  | **>3.5** | 4 (25.0) | 12 (42.9) | 16 (36.4) |  |
| **LDH *(IU/L)*** | **≤ 250** | 13 (81.3) | 2 (7.1) | 15 (34.1) | **< 0.001 *** |
|  | **>250** | 3 (18.8) | 26 (92.9) | 29 (65.9) |  |
| **β2M** | **Negative** | 0 (0.0) | 1 (3.6) | 1 (2.3) | 0.444 |
|  | **Positive** | 16 (100) | 27 (96.4) | 43 (97.7) |  |
| **SPE** | **IgG kappa** | 9 (56.3) | 13 (46.4) | 22 (50.0) | 0.448 |
|  | **IgG lambda** | 3 (18.8) | 9 (32.1) | 12 (27.3) |  |
|  | **IgA kappa** | 3 (18.8) | 2 (7.1) | 5 (11.4) |  |
|  | **IgA lambda** | 1 (6.3) | 4 (14.3) | 5 (11.4) |  |
| **FLC** | **Kappa-LC** | 2 (12.5) | 1 (3.6) | 3 (6.8) | 0.506 |
|  | **Lambda-LC** | 2 (12.5) | 3 (10.7) | 5 (11.4) |  |
|  | **No FLC** | 12 (75.0) | 24 (85.7) | 36 (81.8) |  |

P-value > 0.05: Non significant; P-value < 0.05: Significant; P-value < 0.01: Highly significant (Chi-square test)

*** OR = 56.3 (95CI: 8.35 – 380.1); Sig. Pos. Risk**

**S4 Table (2): The association of mTOR and other parameters:**

|  | | **mTOR score level** | | **Total**  **No. (%)** | **P**  **value** |
| --- | --- | --- | --- | --- | --- |
|  |  | **> 1 (n= 27)** | **≤ 1 (n= 17)** |  |  |
|  |  | **No. (%)** | **No. (%)** |  |  |
| ***Demographic data*** | | | | | |
| **Sex** | **Females** | 10 (37.0) | 7 (41.2) | 17 (38.6) | 0.784 |
|  | **Males** | 17 (63.0) | 10 (58.8) | 27 (61.4) |  |
| **Age *(years)*** | **≤ 35** | 15 (55.6) | 10 (58.8) | 25 (56.8) | 0.831 |
|  | **>35** | 12 (44.4) | 7 (41.2) | 19 (43.2) |  |
| ***Hematological data*** | | | | | |
| **HB *(gm/dl)*** | **< 10** | 21 (77.8) | 14 (82.4) | 35 (79.5) | 0.714 |
|  | **≥ 10** | 6 (22.2) | 3 (17.6) | 9 (20.5) |  |
| **BMA plasma cells *(%)*** | **≤ 36** | 18 (66.7) | 11 (64.7) | 29 (65.9) | 0.894 |
|  | **>36** | 9 (33.3) | 6 (35.3) | 15 (34.1) |  |
| **BMB CD 138 *(%*)** | **≤80** | 13 (48.1) | 11 (64.7) | 24 (54.5) | 0.283 |
|  | **>80** | 14 (51.9) | 6 (35.3) | 20 (45.5) |  |
| **BMB pattern of distribution** | **Diffuse** | 17 (63.0) | 11 (64.7) | 28 (63.6) | 0.907 |
|  | **Interstitial** | 10 (37.0) | 6 (35.3) | 16 (36.4) |  |
| ***Biochemical profile*** | | | | | |
| **Creatinine *(mg/dl)*** | **≤ 2** | 17 (63.0) | 10 (58.8) | 27 (61.4) | 0.784 |
|  | **>2** | 10 (37.0) | 7 (41.2) | 17 (38.6) |  |
| **Ca *(mg/dl)*** | **≤ 11** | 21 (77.8) | 12 (70.6) | 33 (75.0) | 0.592 |
|  | **>11** | 6 (22.2) | 5 (29.4) | 11 (25.0) |  |
| **Albumin *(gm/dl)*** | **≤ 3.5** | 17 (63.0) | 11 (64.7) | 28 (63.6) | 0.907 |
|  | **>3.5** | 10 (37.0) | 6 (35.3) | 16 (36.4) |  |
| **LDH *(IU/L)*** | **≤ 250** | 2 (7.4) | 13 (76.5) | 15 (34.1) | **< 0.001*** |
|  | **>250** | 25 (92.6) | 4 (23.5) | 29 (65.9) |  |
| **β2 microglobulin** | **Negative** | 1 (3.7) | 0 (0.0) | 1 (2.3) | 0.422 |
|  | **Positive** | 26 (96.3) | 17 (100) | 43 (97.7) |  |
| **SPE** | **IgG kappa** | 13 (48.1) | 9 (52.9) | 22 (50.0) | 0.597 |
|  | **IgG lambda** | 8 (29.6) | 4 (23.5) | 12 (27.3) |  |
|  | **IgA kappa** | 2 (7.4) | 3 (17.6) | 5 (11.4) |  |
|  | **IgA lambda** | 4 (14.8) | 1 (5.9) | 5 (11.4) |  |
| **FLC** | **Kappa-LC** | 2 (5.4) | 1 (14.3) | 3 (6.8) | **0.074** |
|  | **Lambda-LC** | 4 (10.8) | 1 (14.3) | 5 (11.4) |  |
|  | **No FLC** | 31 (83.8) | 5 (71.4) | 36 (81.8) |  |

*P-value > 0.05: Non significant; P-value < 0.05: Significant; P-value < 0.01: Highly significant (Chi-square test).

**S4 Table (3): Overall Survival (OS) of the studied patients and its relation to other prognostic factors:**

| Parameter |  | Total No. | Median Survival Time  (months) | No. of Events | Cumulative Survival at 12 months (%) | Cumulative Survival at 24 months (%) | P value |
| --- | --- | --- | --- | --- | --- | --- | --- |
| Whole group |  | 44 | 20.5 | 10 | 38.6 | 38.6 |  |
| Age *(years)* | <53 | 25 | 18 | 5 | 64.70 | 52.90 | 0.696 |
|  | >53 | 19 | 21 | 5 | 35.30 | 47.10 |  |
| Sex | Female | 17 | 25 | 3 | 29.40 | 52.90 | 0.302 |
|  | Male | 27 | 18 | 7 | 70.60 | 47.10 |  |
| HB (gm/dl) | <10 | 35 | 20 | 7 | 82.40 | 82.40 | 0.696 |
|  | >10 | 9 | 22 | 3 | 17.60 | 17.60 |  |
| BMA PC *(%)* | <36 | 29 | 21 | 8 | 58.80 | 64.70 | 0.529 |
|  | >36 | 15 | 20 | 2 | 41.20 | 35.30 |  |
| BMB CD138 *(%)* | <80 | 24 | 22 | 5 | 52.90 | 58.80 | 0.893 |
|  | >80 | 20 | 15.5 | 5 | 47.10 | 41.20 |  |
| BMB Pattern | Diffuse | 28 | 19.5 | 8 | 52.90 | 64.70 | 0.367 |
|  | Interstitial | 16 | 21 | 2 | 47.10 | 35.30 |  |
| Creatinine *(mg/dl)* | <2 | 27 | 20 | 6 | 70.60 | 52.90 | 0.569 |
|  | >2 | 17 | 21 | 4 | 29.40 | 47.10 |  |
| Calcium *(mg/dl)* | <11 | 33 | 22 | 7 | 70.60 | 82.40 | 0.67 |
|  | >11 | 11 | 15 | 3 | 29.40 | 17.60 |  |
| Albumin *(gm/dl)* | <3.5 | 28 | 20.5 | 6 | 58.80 | 70.60 | 0.747 |
|  | >3.5 | 16 | 19 | 4 | 41.20 | 29.40 |  |
| LDH *(IU/L)* | <250 | 15 | 39 | 1 | 17.60 | 64.70 | **0.003*** |
|  | >250 | 29 | 16 | 9 | 82.40 | 35.30 |  |
| β2.microglobulin  *(mg/l)* | ≥ 3.5 | 1 | 11 | 0.1 | 0.0 | 0.0 | 0.176 |
|  | < 3.5 | 43 | 21 | 0.9 | 100 | 100 |  |
| SPEP | IgA Kappa | 5 | 26 | 0.1 | 5.90 | 17.60 | 0.263 |
|  | IgA lambda | 5 | 21 | 0.0 | 23.50 | 5.90 |  |
|  | IgG kappa | 22 | 21.5 | 0.4 | 52.90 | 52.90 |  |
|  | IgG lambda | 12 | 15.5 | 0.5 | 17.60 | 23.50 |  |
| FLC | Kappa-LC | 3 | 37 | 0.0 | 5.90 | 11.80 | 0.831 |
|  | Lambda-LC | 5 | 17 | 0.1 | 11.80 | 11.80 |  |
|  | No FLC | 36 | 20.5 | 0.9 | 82.40 | 76.50 |  |
| RGS1 | ≤ 35 | 16 | 39 | 0 | 11.80 | 82.40 | **0.001*** |
|  | > 35 | 28 | 15 | 10 | 88.20 | 17.60 |  |
| mTOR | >1 | 27 | 15 | 10 | 76.50 | 23.50 | **0.002*** |
|  | ≤1 | 17 | 39 | 0 | 23.50 | 76.50 |  |

*P-value > 0.05: Non significant; P-value < 0.05: Significant; P-value < 0.01: Highly significant (Chi-square test)

**S4 Table (4): Multivariate analysis for OS (cox regression model):**

|  | **Beta coeffecient** | **Stander error** | **P value** | **Hazard ratio** | **95.0% CI for Hazard ratio** | |
| --- | --- | --- | --- | --- | --- | --- |
|  | | | | | **Lower** | **Upper** |
| **LDH** | 0 | 0.001 | 0.822 | 1 | 0.998 | 1.002 |
| **RGS1** | 0.025 | 0.013 | **0.05*** | 1.025 | 1 | 1.05 |
| **mTOR** | 0.316 | 0.122 | **0.01*** | 1.372 | 1.079 | 1.744 |

CI: confidence interval.
